# Systems bioengineering approaches for developmental toxicology

**DOI:** 10.1016/j.csbj.2023.06.005

**Published:** 2023-06-07

**Authors:** Beatriz Xavier Soares, Cláudia C. Miranda, Tiago G. Fernandes

**Affiliations:** a Department of Bioengineering and iBB – Institute for Bioengineering and Biosciences, Instituto Superior Técnico, Universidade de Lisboa, Lisbon, Portugal; b Associate Laboratory i4HB – Institute for Health and Bioeconomy, Instituto Superior Técnico, Universidade de Lisboa, Lisbon, Portugal; c AccelBio, Collaborative Laboratory to Foster Translation and Drug Discovery, Cantanhede, Portugal

**Keywords:** Developmental toxicology, Systems bioengineering, Human pluripotent stem cells, High-throughput screening, Mathematical modeling

## Abstract

Developmental toxicology is the field of study that examines the effects of chemical and physical agents on developing organisms. By using principles of systems biology and bioengineering, a systems bioengineering approach could be applied to study the complex interactions between developing organisms, the environment, and toxic agents. This approach would result in a holistic understanding of the effects of toxic agents on organisms, by considering the interactions between different biological systems and the impacts of toxicants on those interactions. It would be useful in identifying key biological pathways and mechanisms affected by toxic agents, as well as in the development of predictive models to assess potential risks of exposure to toxicants during development. In this review, we discuss the relevance of systems bioengineering to the field of developmental toxicity and provide up-to-date examples that illustrate the use of engineering principles for this application.

## 1 Introduction

A systems bioengineering approach to studying developmental toxicity aims to comprehend the intricate molecular, cellular, and physiological processes involved in the adverse effects of toxicants during embryonic development. This approach combines techniques from engineering (such as systems and control theory [1] and biology/toxicology to give a complete understanding of toxicity at various biological organization levels [2]. The key advantage of this approach is the ability to predict chemical toxicity before testing in animals or humans, therefore reducing animal testing, and enhancing human safety [3]. This systems approach can also reveal the specific molecular pathways and physiological processes affected by toxicants, offering new targets for safer chemical development [4].

**Fig. 1:**
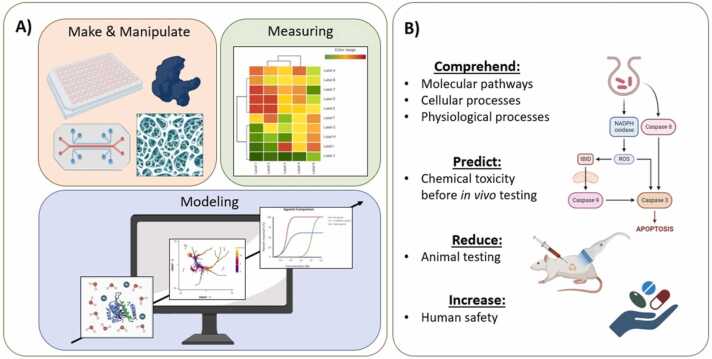
**A)** The three pillars of systems bioengineering: make, and/or manipulate artificial cellular niches *in vitro*; measuring events in such environments; and building predictive *in silico* models. **B)** The systems bioengineering approach to developmental toxicity offers several advantages, including the identification of specific molecular pathways and physiological processes, the ability to predict toxicity before animal testing, a multi-level analysis of toxicity, and an improved understanding of the complex mechanisms involved in developmental toxicity. By combining systems biology and bioengineering principles, this approach allows for a comprehensive understanding of how toxicants can affect developing organisms at multiple levels of biological organization.

The term 'Systems Bioengineering' was first used by Lauffenburger and co-workers when trying to articulate an all-inclusive definition for the increasing number of cases where elements of engineering science were used in the study of biological systems [5]. This blend of engineering and biological techniques is grounded in three pillars: the ability to create, recreate, and/or manipulate artificial cellular niches *in vitro*; the capacity to measure events happening in such environments with increasing resolution; and the capability to build predictive *in silico* models based on collected data and theoretical frameworks [6], [7] (Fig. 1**A**). In the case of developmental toxicology, this approach may contribute to a comprehensive and predictive understanding of toxicity, reducing the need for animal testing and improving human health and safety. Examples of studies utilizing this approach include the use of organoids or microfluidic devices to recreate cellular and physiological interactions in embryonic development [8], and computational models to simulate toxicant effects on these interactions [9].

These studies have provided valuable insights into developmental toxicity mechanisms and improved our understanding of the complex processes involved. Systems bioengineering can therefore provide a way to enhance our understanding of the harmful effects of different molecules and help developing safer chemicals.

In fact, the combination of systems biology, bioengineering, and computational modeling allows a comprehensive understanding of the interactions between biological systems and the environmental factors that can lead to toxic effects. One of the key advantages of this approach is the ability to predict toxicity before animal testing [10]. Traditional methods of toxicity testing, which rely on animal models, are often time-consuming, expensive, and ethically challenging [11]. By contrast, systems bioengineering offers the potential for more efficient and accurate toxicity screening, allowing researchers to identify potential hazards and prioritize further testing and research. This not only reduces the need for animal testing but also offers a more comprehensive understanding of toxicity. Furthermore, a systems bioengineering approach enables the identification of specific molecular pathways and physiological processes that are affected by developmental toxicity [12]. By analyzing gene expression data and protein networks, for example, researchers can identify the underlying biological mechanisms [13]. This knowledge can be utilized to develop more precise interventions and to enhance the safety of chemicals and products.

Another advantage of a systems bioengineering approach is the importance of multilevel analysis of toxicity. Developmental toxicity is a complex process that involves interactions at multiple levels of biological organization, including the genome, proteome, and metabolome [14], [15]. By integrating data from these different levels, systems bioengineering provides a more comprehensive and integrated understanding of the mechanisms of toxicity. Finally, by modeling the interactions between environmental factors and biological systems, researchers can gain new insights and develop new hypothesis for the underlying biological processes.

It is therefore possible to conclude that a systems bioengineering approach to developmental toxicity offers numerous advantages, including the ability to predict toxicity before animal testing, the identification of specific molecular pathways and physiological processes, a multilevel analysis of toxicity, and an improved understanding of the complex mechanisms involved in developmental toxicity (Fig. 1**B**). By combining different approaches, researchers can gain more detailed knowledge and develop targeted interventions to prevent developmental toxicity. In the following sections, we provide further details and highlight relevant examples that offer proof-of-concept evidence of the importance of this approach.

## 2 Building *in vitro* models of development

**Fig. 2:**
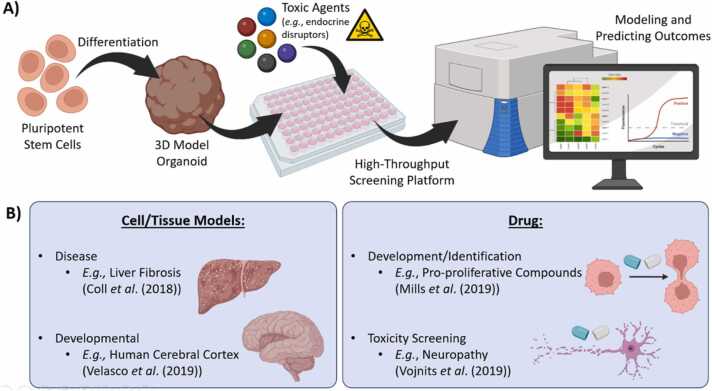
**A)** One example of the application of the systems bioengineering concept is in development toxicology, where human pluripotent stem cells can be used to build *in vitro* models of development, such as organoids. These models can be integrated into high-throughput screening platforms with the objective of assessing the toxicity of different agents. Finally, based on the generated data (*e.g.*, omics), mathematical modeling can be employed to generate predictions of toxicity outcomes. **B)** This paradigm can be used to develop models of different tissues and interrogate how they are formed (*e.g.*, the cerebral cortex), or how they are affected by pathological processes (*e.g.*, liver fibrosis). Such models can also be used to assist in the identification of new compounds or screen for their toxic effects.

Building *in vitro* models of development is essential for studying the early stages of cell differentiation and the effects of various substances during this process (Fig. 2**A**). In fact, the widespread use of animal models has several limitations, including inaccuracies and ethical concerns regarding animal cruelty. Cell culture was firstly established using two-dimensional culture systems, which are still widely used today for preliminary results due to their simplicity. However, these models also have limitations when it comes to simulating *in vivo* environments. [16]. They differ from *in vivo* conditions in terms of morphology, proliferation rates, differentiation potentials, cell interactions with each other and the surrounding matrix, and signal transduction [16]. Three-dimensional culture systems, however, more accurately mimic *in vivo* conditions, bridging some of the gaps between biological processes that occur within a living organism, and those that occur in the laboratory. These models can be adapted to study the development of various tissues and their behavior after maturation. In this context, *in vitro* models of development are primarily derived from human pluripotent stem cells (PSCs), and their differentiation can recreate the formation of the trophoblast [17], simulate gastrulation events [18], or generate cells from the three germ layers [19], [20], [21].

The blastocyst consists of trophoblast cells that can differentiate into all cells of the extra-embryonic tissues, and the inner cell mass, which specializes into the ectoderm, mesoderm, and endoderm. Studying the development and organization of these cells is challenging due to the difficulties in obtaining embryos at early stages of development and the ethical concerns surrounding their use in research. Therefore, it is important to develop models that accurately depict the development of these tissues and their response to stimuli such as drugs and pathogens. Karvas et al. (2022) developed a stem-cell-derived trophoblast organoid model that represents human placental development and was found to be suitable for testing the tissue susceptibility to pathogens [17]. Additionally, gastrulation represents the process in which an embryo transforms from a single-layer blastula into a multi-layered and three-dimensional gastrula structure [22]. Using embryonic stem cells (ESCs), Moris et al. (2020) generated gastruloids to model the regulatory processes during early anteroposterior organization by incubating human ESCs with medium supplemented with Chiron, a WNT antagonist [18]. On the other hand, Minn et al. (2020) demonstrated that culturing ESCs with bone morphogenetic protein 4 (BMP4) resulted in the differentiation of cells into gastruloids, which expressed specific markers of the three germ layers and extra-embryonic cells, organized in a radial pattern, accurately mimicking specific features of embryogenesis [23]. In another example, Olmsted and Paluh (2022) used human induced pluripotent stem cells (iPSCs) to develop a gastruloid model of combined cardio- and neurogenesis [24]. Cells were cultured for two days in medium supplemented with fibroblast growth factor 2 (FGF2) and CHIR99021 (a GSK-3 inhibitor), followed by three days in medium with insulin growth factor 1 (IGF1) and hepatocyte growth factor (HGF), and finally two additional days with FGF2 and vascular endothelial growth factor (VEGF) supplementation.

For a more specialized approach, models for specific tissues derived from one of the germ layers can also be developed. The extensive repertoire of organoids that can be created to model various organ systems and developmental stages is beyond the scope of this paper and has been comprehensively reviewed elsewhere [25]. However, the types of organoids that have been successfully developed and have applicability in developmental toxicology include liver, lung, gut, pancreas, kidney, skeletal muscle, brain, retina, and vascular organoids, among others [26].

For example, the creation of human brain organoids is crucial for studying brain development and diseases. In fact, Velasco et al. (2019) outlined a protocol that could generate low-variability organoids of the dorsal forebrain including various cell types from the human cerebral cortex, through culture medium supplementation with IWR1 (a WNT inhibitor) and SB431542 (a TGFβ inhibitor) for 18 days, followed by exposure to heparin from day 35–56 of culture [19]. Following this, Giandomenico et al. (2021) provided an enhanced protocol to generate cerebral organoids capable of long-term culture, which allows the study of later stages of development such as axon growth and neuronal maturation [27]. This is achieved through modification of the embryoid bodies shape and size. All these efforts are based on previous work by Lancaster and Knoblich (2014) [28], which involved using small embryoid body culture in medium supplemented with low levels of FGF2 for four days, followed by culture with neural induction medium supplemented with heparin for the same period. Cerebral organoid differentiation could then be induced for two days with a medium supplemented with vitamin A, and two days in its absence. This supplementation scheme promoted growth of cerebral tissue and allowed the organoids to be maintained in culture for up to a year.

Several models of heart tissue development have also been proposed to improve current methodologies by mimicking *in vivo* biological processes. Yang et al. (2022) [20], for example, outline a multi-lineage heart model that provides cardiomyocytes from the first heart field (FHF) through increased stimulation with BMP4 and nodal signaling. For anterior and posterior second heart field (aSHF, and pSHF) generation, however, lower levels are needed. On a different model, Coll et al. (2018) described a protocol for the *in vitro* production of liver spheroids capable of modeling hepatic development, liver fibrosis, and that could be used for drug toxicity screening [21]. For this protocol, human PSCs were cultured in hepatic differentiation medium supplemented with BMP4 for four days, followed by FGF1 and FGF3 stimulation for additional four days. Retinol and palmitic acid were also supplemented from day 6 to 12. The resulting non-parenchymal hepatic stellate cells were then co-cultured with primary hepatic stem cells (HSCs) obtained from non-tumor liver tissue to generate 3D liver spheroids. This created a suitable system for modeling different liver disorders and developing new screening platforms.

More recently, multi-lineage models have been developed involving a combination of cells from different germ layers that are co-generated and mature side by side. Branco et al. (2022) provide an example of this phenomenon with a meso- and endoderm co-culture [29]. In this study, the authors describe a protocol for the generation of an epicardium/myocardium organoid supported by pro-epicardium/foregut tissues. These complex structures recreate the natural tissue organization that occurs during *in vivo* embryogenesis. The protocol starts by culturing human PSCs in medium with CHIR99021 supplementation in the absence of insulin for three days, and then with WNT inhibition for two additional days. This results in the generation of cardiomyocyte aggregates. In parallel, to recreate the pro-epicardium, septum transversum mesenchyme, and posterior foregut, human PSCs were cultured for two days in medium supplemented with CHIR99021, BMP4, and retinoic acid. By co-culturing these spheroids with cardiomyocyte aggregates, the authors could generate a self-organized heart organoid comprising an epicardium-like layer that fully surrounds a myocardium-like tissue. Silva et al. (2021) took further advantage of this cooperation between cardiac and gut tissue by following a similar approach [30]. Yet another multi-lineage cell-model was proposed by Ng et al. (2022) to study the development and interaction of human cardiac and pulmonary tissues during embryogenesis by simultaneously inducing meso- and endoderm through WNT and nodal signaling [31]. It is also possible to study the development of multiple tissues within a neuronal network by using a combination of meso- and ectoderm-derived cells. Martins et al. (2020) describe a protocol for the formation of self-organizing neuromuscular organoids with the potential to model neuromuscular diseases [32]. This feature was achieved by using human PSC-derived axial stem cells to simultaneously generate spinal cord neurons and skeletal muscle cells that self-organize to generate neuromuscular organoids. These structures could be maintained for several months and were capable to recapitulate key aspects of neuromuscular networks.

In conclusion, organoids have garnered significant interest in toxicology research due to their unique advantages, although they are not without limitations. As mentioned previously, one of the major strengths of organoids is their ability to closely resemble human tissue. By using human cells, organoids replicate the structural and functional characteristics of specific organs, allowing researchers to study toxicity with greater human relevance compared to traditional animal testing. Furthermore, organoids facilitate high-throughput screening. Another advantage lies in their potential for disease modeling. By deriving organoids from patient-specific cells, researchers can recreate specific diseases or genetic disorders in the laboratory setting. This personalized approach enables the investigation of toxicity on diseased tissues, leading to a deeper understanding of how drugs or environmental compounds may affect individuals with specific conditions. This disease modeling aspect has the potential to contribute to the development of personalized medicine and tailored treatment strategies. From an ethical standpoint, by reducing the reliance on animal models, organoids address the ethical concerns associated with traditional testing methods, aligning with the principles of the 3Rs (Replacement, Reduction, and Refinement).

However, despite their advantages, there are certain limitations to consider when using organoids in toxicology studies. One key limitation is the complexity of organoids. While they can mimic certain aspects of organ structure and function, they may not fully replicate the intricate interactions and complexities of the whole organ system. Additionally, there can be inherent variability between organoids generated from different individuals or batches. This variability can affect experimental outcomes and make it challenging to establish consistent toxicity screening protocols or compare results across different studies. Efforts to standardize protocols and develop validation criteria are thus necessary to ensure reproducibility and reliability of organoid-based toxicology research. Another limitation is the maturity and functionality of organoids. Organoids often do not fully mature or reach the same level of functionality as their *in vivo* counterparts. This immaturity can impact their response to toxicants, potentially leading to incomplete or inaccurate toxicity assessments.

In summary, while organoids hold tremendous potential in toxicology research, there are several considerations to keep in mind. Their ability to provide human relevance, enable high-throughput screening, and facilitate disease modeling are significant advantages. However, the limitations related to complexity, variability, maturity, and functionality need to be addressed through standardization efforts and further research.

## 3 Screening developmental toxicology

High-throughput screening models for developmental toxicology can be designed through the combination of microscale platforms and omics. A variety of these models have been developed, including some of the examples mentioned in the previous section (Fig. 2**B**). Additionally, Mills et al. (2019) showcased a human cardiac organoid platform that allows for the identification and development of pro-regenerative drugs [33]. According to this protocol, the organoids were cultured and matured in a postnatal metabolic environment in 96-well plates, minimizing the need for tissue handling and providing a reliable platform for testing various compounds. This platform enabled real-time analysis of compound side effects in cell proliferation and functionality, as well as their mechanisms of action through RNA sequencing and proteomic profiling. Czerniecki et al. (2018) described a fully automated high-throughput screening platform for differentiation and phenotyping of human kidney organoids [34], particularly using immunofluorescence through staining cells with *Lotus tetragonolobus* lectin (capable of binding to the proximal tubular segments), and RNA sequencing. This automated protocol relied on the use of 96- or 384-well plates and a one-step differentiation protocol, through the exposure of cells to CHIR99021. Another model utilizing kidney cells was developed by Tran et al. (2022) for modeling polycystic kidney disease and drug discovery using transcriptomic profiling and immunofluorescence [35]. An adapted differentiation protocol was used, consisting of a 2D cell culture of human PSCs in medium supplemented with CHIR99021 for four days, Activin-A for three days, and an additional day with FGF9. This was followed by 3D culture in micro-fabricated plastic vessels used for mass-production of aggregates with uniform size, in medium supplemented with CHIR99021 and FGF9 for two days. The medium was then changed to RPMI 1640 supplemented with FGF9 for the remainder of the culture. These controlled sized organoids could then be immobilized in methylcellulose 96-well plates for further culture and imaging.

On a different note, Yan et al. (2018) advanced a human gastric cancer organoid biobank comprising healthy cells and various subtypes of cancer cells for disease modeling and drug screening through transcriptome analysis [36]. Other models have also been developed for evaluating toxic side effects, like the platform described by Vojnits et al. (2019) [37], which used sensory neurons derived from adult peripheral blood cells to study peripheral neuropathy, a condition that results from damage to the peripheral nervous system following chemotherapy. This protocol starts with the selection and expansion of CD34^+^ mononuclear cells from peripheral blood donor samples. These cells were then induced to neural progenitor cells through a 24-hour exposure to expansion medium supplemented with SB431542, LDN193189, CHIR99021, and a Lenti-virus OCT4 delivery system. Two days after infection, the cells were cultured with DMEM/F12 with FGF2 and knockout serum replacement for three days, followed by culture with neural precursor medium. Differentiation was achieved by culturing the cells in a sensory neuron specification medium for one to two weeks. The resulting sensory neurons were then used for drug testing based on immunofluorescence, flow cytometry, cell viability, and calcium imaging.

Finally, one example of a microfluidic system is the blood-brain barrier chip developed by Vatine et al. (2019) [36], using organ-on-chip technology and brain microvascular endothelial-like cells, astrocytes, and neurons derived from induced PSCs, that was capable of modeling neurological disorders for personalized medicine and drug screening applications [38]. Also starting from human PSCs, another study demonstrated the formation of neural aggregates with structures resembling neural rosettes in a microwell array [39]. This platform was used to study the effects of neurotoxic molecules on embryonic development, as demonstrated by the analysis of the teratogenic potential of the antiepileptic drug valproic acid.

Overall, microfluidic technology can be used to generate integrated organ-on-chip platforms designed to better mimic human physiology, control cell microenvironments, and maintain tissue-specific functions [40]. These multi-organoid approaches are becoming more common in the field, with the expectation that they can help recreate the natural niche, study complex interactions between different organoids, and more accurately mimic physiological conditions [41]. It would also be relevant to include environmental control elements and the delivery of biomimetic stimuli that drive physiological responses [42]. For example, it is possible to test developmental signals originating from outside the cellular niche using microfluidic technology. The endocrine system is particularly important for controlling tissue differentiation and homeostasis. A microfluidic platform that supports ovarian follicles and is capable of simulating the menstrual cycle hormone profile has been shown to replicate the female reproductive tract and the endocrine loops between the ovary, fallopian tube, uterus, cervix, and liver, with sustained circulating flow between all tissues [43]. This represents a powerful tool that allows organ-organ integration of hormonal signaling and has great potential for use in drug discovery and toxicology studies. Recognizing the importance of these external factors in regulating niche cells is indeed crucial in understanding the broader context of biosystems engineering. Furthermore, integrated microfluidic platforms may also enable predictions of drug absorption, distribution, metabolism, excretion, and toxicity. One model with gut, liver, and kidney compartments for simulating orally administered drugs, and vascular, liver, and kidney compartments for intravenous administration, can be used to replicate absorption, distribution, hepatic activation, and excretion [44]. This capability is critical for understanding toxicity and evaluating the potential adverse effects of chemicals on human health.

Still, it is important to acknowledge that while these approaches can reduce the reliance on animal testing, there are instances where animal studies remain necessary to validate and further investigate the findings obtained from *in vitro* models. Therefore, the ultimate goal of systems bioengineering is to minimize and refine animal usage. However, at the present stage of scientific advancement, animal testing continues to play an integral role in regulatory toxicology and risk assessment. Nevertheless, ongoing efforts are being made to develop alternative testing methods, including *in silico* modeling, with the aim of further reducing the reliance on animal studies in the future.

## 4 Mathematical modeling of the risk of exposure to toxicants during development

The final pillar of systems bioengineering is the capacity to build predictive *in silico* models based on collected data and theoretical frameworks. In fact, mathematical modeling has become a powerful tool for understanding and predicting the risks associated with exposure to toxicants during development [45]. Since the human body is constantly exposed to a variety of chemicals, radiation, and biological agents, exposure to such agents during development can have long-lasting effects on health and wellbeing, including developmental delays, birth defects, and increased risk of chronic diseases later in life [46]. Therefore, the use of mathematical equations to describe the relationships between exposure levels, toxicant concentrations, and health outcomes is very valuable. This approach allows scientists to explore complex systems and predict how different factors may interact to produce a particular outcome. In the case of toxicant exposure during development, mathematical modeling can help to identify the factors that contribute to risk, as well as the most effective strategies for reducing that risk.

One important application of mathematical modeling is in predicting perturbations to normal embryogenesis. Gastrulation is a crucial process in the early stages of mammalian embryo development, transforming a group of cells into organized tissues with reference to three orthogonal axes. Recent research has shown that human PSCs can be used to generate gastruloids, which are 3D multicellular aggregates that differentiate to form derivatives of the three germ layers [18]. These gastruloids exhibit spatiotemporal organization and elongation along an anteroposterior axis, with patterned gene expression, including a signature of somitogenesis. To further study the positional-dependent cell fate acquisition during gastrulation, a system was developed using human PSCs [47]. This method induced gastrulation-associated patterning in geometrically confined colonies. Upon BMP4 treatment, phosphorylated SMAD1 (pSMAD1) activity in the colonies organized into a radial gradient, consistent with a reaction-diffusion model. Consequent fate acquisition occurred as a function of both pSMAD1 signaling strength and duration of induction, consistent with the positional-information paradigm. This experimental information was organized into a two-step mathematical model that predicted responses to key perturbations. Furthermore, it also predicted experimental conditions that resulted in reaction-diffusion patterning in large human PSC colonies and rescued peri-gastrulation-like patterning in colony sizes previously thought to be reticent to this behavior. Additionally, biomechanics can also play significant roles in embryonic development, and models of neuroectoderm development have been established in which geometrical confinement induced patterning of cells, mimicking regionalization observed during *in vivo* neurulation [48].

Human PSCs are thus capable of self-organizing into multicellular organoids that mimic the complex tissue development of the human body. However, controlling the self-directed pattern formation of these organoids remains a significant challenge. Recent research has combined genetic engineering with computational modeling, machine learning, and mathematical pattern optimization to develop a data-driven approach to control cell self-organization [49], [50]. Researchers used CRISPR interference to knockdown genes CDH1 and ROCK1, which affect stem cell colony organization. They then replicated experimental phenomena *in silico* using an extended cellular Potts model and optimized model parameters using machine learning. The predicted outcomes were then validated *in vitro*, demonstrating that morphogenic dynamics can be accurately predicted through model-driven exploration of cell behaviors *via* machine learning. This approach enabled mathematical modeling of multicellular spatial patterning to guide the generation of human organoids. However, a key aspect in organoid positional modeling is mass transport. Thus, mathematical, and computational approaches to describe and predict organoid dynamics are particularly useful [51]. In another example, mathematical models were employed to study key metabolite gradient distributions (*e.g.*, glucose and lactate), which are critical for the growth and maintenance of organoids [52]. These models were used to predict the evolution of the spatial metabolite distributions, as well as metrics quantifying glucose conversion, maximum lactate concentration, and the likelihood of intolerable lactate concentrations.

Another application of mathematical modeling is in the assessment of cumulative risk. Cumulative risk refers to the combined effects of exposure to multiple toxicants, which can have non-linear (*e.g.*, additive, or synergistic) effects on health outcomes. Mathematical models can help to predict the cumulative effects of exposure to different toxicants, taking into account factors such as the timing and duration of exposure, the potency of each toxicant, and the individual characteristics of the exposed cell population [9], [53]. Furthermore, recent advances in single-cell analysis raise the possibility to interrogate potential toxic effects of compounds at the single-cell level. Complex phenomena like cell fitness and competition, in particular, may explain why certain cell populations are lost, while others become dominant. Such 'loser' cells vanish by direct elimination or by limited access to survival factors [54]. Therefore, mathematical models can simulate complex cell competition in multicellular dynamic systems. For instance, embryonic tissue patterning, and lineage commitment of human PSCs based on gene regulatory networks (GRNs) can be modeled in response to dynamic microenvironments. One such model is the GARMEN strategy, which utilizes a GRN model of human PSC lineage commitment embedded in cellular agents [55]. The simulations using GARMEN demonstrated the significant impact of GRN wiring on tissue pattern order, composition, and dynamics. Moreover, experimental perturbation of GRN connectivity confirmed the model predictions and highlighted the role of OCT4 as a master regulator of peri-gastrulation fates. Another computational platform, IQCELL, offers a versatile tool to infer, simulate, and study executable GRNs in dynamic biological systems. IQCELL utilizes single-cell RNA-sequencing data from various developmental systems to infer overall causal gene interactions [56]. Dynamic simulations of the generated GRNs can recapitulate the effects of known gene perturbations and identify candidate genes without prior knowledge. Hence, these models provide novel opportunities to understand complex interactions between morphogenetic signals, GRNs responsible for tissue patterning and lineage commitment, and eventually predict how perturbations to the system will impact the outcome of such developmental processes.

On a different note, computational modeling may also be used in the development of exposure guidelines and risk assessments [57]. Exposure guidelines are established to protect individuals from harmful levels of exposure to toxicants, while risk assessments are used to evaluate the likelihood of adverse health effects associated with exposure at different levels. Mathematical models can help establish safe exposure levels based on a range of factors, including the potency of the toxicant, the size and characteristics of the exposed population, and the duration and frequency of exposure. One particular area of research that has benefited greatly from mathematical modeling is the study of endocrine disruptors (EDCs) [58]. Endocrine disruptors are chemicals that interfere with the normal functioning of hormones, which can have significant effects on development and health [59], [60]. Mathematical models have helped to elucidate the complex interactions between endocrine disruptors and the endocrine system [61], as well as the long-term health effects of exposure. Computer-aided technologies may use quantitative structure-activity relationship methods, which correlate the chemical structure of compounds with biological activity through molecular descriptors [62]. With the increasing number of three-dimensional crystal structures of EDCs' targets, docking and scoring procedures can be used to predict their interactions. These computer-assisted approaches provide a valuable tool for predicting endocrine disruptor activities and promoting public health.

## 5 Conclusion

By using principles of systems biology and bioengineering, it is possible to gain further insights into the complex mechanisms that influence developing organisms and understand how perturbations affect normal biological processes. In the case of developmental toxicology, the key advantage of this approach is the ability to predict chemical toxicity before testing in animals or humans, reducing animal testing, and enhancing human safety. Still, the translational potential of systems bioengineering approaches extends beyond human biology. The principles and techniques discussed in our article can be adapted and utilized to study developmental toxicity in various animal models, such as rodents, zebrafish, or invertebrates like *Drosophila* or *C. elegans*. Therefore, applying systems bioengineering approaches may reveal both the similarities and differences in developmental processes among species. Moreover, these tools can be further explored in environmental toxicology, where understanding the impact of chemicals on non-human organisms and ecosystems is crucial. The integration of ecological models and bioengineering techniques may contribute in the future to comprehending the effects of developmental toxicants on wildlife populations and ecological systems.

Screening platforms for developmental toxicology can be constructed with the objective of evaluating potential impacts of toxicants in *in vitro* models of development. A variety of such models have been developed through a combination of microscale platforms and omics. Significant examples include models of gastrulation and further differentiation into derivatives of the three germ layers. Also of note is the impact of mathematical modeling in developmental toxicology. This approach has become an essential tool in the study of toxicant exposure during development. By allowing scientists to explore complex systems and predict the effects of exposure, mathematical models can help to identify the factors that contribute to risk, establish safe exposure levels, and inform policy decisions aimed at reducing the harmful effects of toxicants on human health. As our understanding of the interactions between toxicants and the human body continues to grow, systems bioengineering will undoubtedly remain a key practice in the fight against exposure-related health risks.

## Conflict of interest

CCM is employed by AccelBio, Collaborative Laboratory to Foster Translation and Drug Discovery, and receives financial compensation as salaried employee. The remaining authors declare no conflict of interests.
